# H_2_O_2_ as a candidate bottleneck for MnP activity during cultivation of *Agaricus bisporus* in compost

**DOI:** 10.1186/s13568-017-0424-z

**Published:** 2017-06-17

**Authors:** Aurin M. Vos, Edita Jurak, Jordi F. Pelkmans, Koen Herman, Gill Pels, Johan J. Baars, Ed Hendrix, Mirjam A. Kabel, Luis G. Lugones, Han A. B. Wösten

**Affiliations:** 1 Microbiology, Department of Biology, Utrecht University, Padualaan 8, 3584 CH Utrecht, The Netherlands; 2 Laboratory of Food Chemistry, Wageningen University, Bornse Weilanden 9, 6708 WG Wageningen, The Netherlands; 3 Plant Breeding, Wageningen University and Research Centre, 6700 AJ Wageningen, The Netherlands

**Keywords:** Fungus, *Agaricus bisporus*, Manganese peroxidase, Hydrogen peroxide, Lignin, Compost

## Abstract

Degradation of lignin by fungi enhances availability of cellulose and hemicellulose in plant waste and thereby increases the amount of carbon source available to these microorganisms. The button mushroom *Agaricus bisporus* degrades only about half of the lignin in compost and about 40% of the carbohydrates remain unutilized during mushroom cultivation. Here it was assessed whether over-expression of the manganese peroxidase gene *mnp1* improves lignin degradation and, as a consequence, carbohydrate breakdown by *A. bisporus*. Transformants expressing *mnp1* under the control of actin regulatory sequences produced MnP activity in malt extract medium, while the parental strain A15 did not. MnP activity was increased 0.3- and 3-fold at casing and after the 2nd flush of a semi-commercial cultivation, respectively, when compared to strain A15. Pyrolysis-GC–MS showed that overexpression of MnP decreased phenylmethane and phenylethane type lignin relative to the phenylpropane type after the 2nd flush. However, it neither affected the syringyl/guaiacyl derived residue ratio nor the ratio of oxidized to non-oxidized lignin residues. Moreover, the carbohydrate content and accessibility was not affected in compost. Notably, the capacity of compost extract to consume the MnP co-factor H_2_O_2_ was 4- to 8-fold higher than its production. This may well explain why over-expression of *mnp1* did not improve carbohydrate degradation in compost. In fact, availability of H_2_O_2_ may limit lignin degradation by wild-type *A. bisporus*.

**Electronic supplementary material:**

The online version of this article (doi:10.1186/s13568-017-0424-z) contains supplementary material, which is available to authorized users.

## Introduction

The button mushroom *Agaricus bisporus* is cultivated worldwide on straw based compost. The commercial substrate of *A. bisporus* used in the Netherlands is produced in two phases of composting. Phase I (PI) occurs in tunnels and comprises a 3–6 day incubation of a mixture of wheat straw, horse manure, gypsum, and water, either or not supplemented with chicken manure (Gerrits 1988). Temperature increases up to 80 °C during this phase, replacing a mesophilic with a thermophilic microbiota (Gerrits 1988). Phase II (PII) starts after the microflora of a previous PII is introduced into PI compost. The temperature is decreased to 50 °C and then increased to 60 °C in a 2-day-period changing the microflora and killing unwanted organisms like insects (Gerrits 1988; Straatsma et al. 1994). This is followed by a 3-day-period at 45 °C during which thermophilic fungi like *Scytalidium thermophilum* (also named *Humicola insolens*; Straatsma and Samson 1993) colonize the compost sequestering ammonia and suppressing competitors of *A. bisporus* (Ross and Harris 1983; Straatsma et al. 1989, 1994). During PI and PII 50–60% of the carbohydrates in the compost is consumed, while lignin remains intact (Jurak et al. 2015). *A. bisporus* is inoculated in PII-end compost. After 16–19 days at 25 °C, about 15, 10, and 50% of xylan, cellulose, and lignin is consumed, respectively, when compared to PII-end (Jurak et al. 2015). Phase IV (PIV) starts by topping the PIII-end compost, either or not supplemented with formaldehyde-treated protein rich substrates, with a casing layer. *A. bisporus* mycelium colonizes this casing layer under high CO_2_ conditions and a relative humidity (RH) of 85%. Mushroom formation is induced by reducing CO_2_ levels, lowering the temperature to 18–22 °C, and increasing RH to 87–90% (Visscher 1988). Mushrooms are produced in 2–3 flushes with 7–8 day intervals, resulting in a typical yield of 30 kg m^−2^ per 85–95 kg compost m^−2^. An additional 44%, 29%, and around 8% of cellulose, xylan, and lignin is degraded, respectively, during PIV (Kabel et al. 2017). Thus, about 20% of the carbohydrates are not consumed when compared to the starting material or 40% relative to PII-end (Iiyama et al. 1994; Chen et al. 2000a; Kabel et al. 2017).

Lignin functions as cohesive between cellulose microfibrils and forms covalent interactions with hemicellulose (Boerjan et al. 2003). Consequently, enzymatic degradation of these carbohydrates is hampered. It has been estimated that lignin removal makes 30% of the carbohydrates available for consumption by *A. bisporus* (ten Have et al. 2003). Lignin consists of the monolignol monomers *p*-coumaryl alcohol, coniferyl alcohol, and sinapyl alcohol (Boerjan et al. 2003; Vanholme et al. 2010) that form *p*-hydroxyphenyl (H), guaiacyl (G), and syringyl (S) phenylpropanoid units within lignin, respectively. Lignin has a heterogeneous and recalcitrant structure that, unlike cellulose and hemicellulose, cannot be degraded by enzymes directly. Therefore, fungi produce lignin peroxidase (LiP), manganese peroxidase (MnP), and versatile peroxidase (VP) that generate extracellular radicals (Kirk and Farrell 1987; Hatakka 1994; ten Have and Teunissen 2001; Guillén et al. 2005). Laccase (Lac) has also been implicated in ligninolysis but their role is less clear (Eggert et al. 1996; 1997; Li et al. 2001).

*Agaricus bisporus* contains 2 *mnp* genes and 13 *lac* genes in its genome (Morin et al. 2012). Manganese peroxidase MnP1 but not MnP2 has been characterized (Bonnen et al. 1994; Lankinen et al. 2001). MnP uses Mn^2+^ and (organic) hydrogen peroxide (H_2_O_2_ or R–OOH) as cofactors (Hofrichter 2002). H_2_O_2_ oxidizes the heme group in MnP to form compound I. Compound I oxidizes Mn^2+^ or a phenolic compound to generate compound II that subsequently oxidizes another Mn^2+^ to return the MnP to its original oxidation state. Oxalic acid chelates the generated Mn^3+^, which stimulates its release from MnP and increases its stability. MnP is able to oxidize both phenolic and non-phenolic lignin model structures. For the latter, other mediators like unsaturated lipids are required to initiate extracellular lipid peroxidation that results in highly reactive compounds. MnP and Lac are highly produced during the vegetative colonization of compost but their activity is reduced during mushroom formation (Wood and Goodenough 1977; Bonnen et al. 1994).

Here, *mnp1* of *A. bisporus* was overexpressed with the aim to improve ligninolytic activity, thereby increasing degradation of hemicellulose and cellulose in compost. Overexpression of *mnp1* increased MnP activity in compost but neither affected lignin content nor carbohydrate content and accessibility. Experimental evidence indicates that this is due to a limiting availability of H_2_O_2_ as a cofactor.

## Materials and methods

### Culture conditions and strains

*Agaricus bisporus* A15 (Sylvan, Kittanning, PA) and its derivatives were grown at 25 °C on malt extract agar medium [MEA; 20 gr l^−1^ malt extract (BD biosciences, Franklin Lakes, USA), 2.1 gr l^−1^ 3-morpholinopropane-1-sulfonic acid, pH 7.0, and 1.5% agar], wheat bran [WB; 4% wheat bran (w/w) in water], or PII-end compost (CNC Grondstoffen, Milsbeek, the Netherlands). Spawn was produced using a mixture of 24 gr kg^−1^ CaSO_4_, 6.87 gr kg^−1^ CaCO_3_, and 75 gr *Sorghum* seeds. The seeds had been heated in water at 100 °C for 20 min followed by sterilization for 20 min at 121 °C. The mixture was colonized for 3 weeks at 25 °C using 2 1-week-old MEA-grown *A. bisporus* colonies as inoculum. PII-end compost was colonized using 18 boxes per strain (40 cm width × 60 cm length × 22 cm height) each containing 16 kg substrate (CNC, Milsbeek, The Netherlands) and each inoculated with 75 gr of spawn. Compost temperature was maintained at 25 °C using an air temperature of 22 °C. RH in the growing room was kept at 95%, while CO_2_ levels were 1500–2000 ppm. Boxes that had been randomly distributed in the growing room (Unifarm, Wageningen) were overlaid with 7 kg casing layer (CNC, Milsbeek, The Netherlands) after 16 days, after which growth was prolonged for 14 days. The casing was manually broken and mixed (ruffled) to create fast regenerative growth and a more equal distribution of *A. bisporus* in the casing layer 4 days prior to venting (i.e. 10 days after casing). Venting resulted in a gradual decrease of compost and air temperature to 19 and 18 °C, respectively, while RH and CO_2_ levels decreased to 85% and 1200 ppm. The first buttons were removed from the bed 9 days after venting. The compost from four boxes of each strain was collected at casing (day 15), venting (day 30), and after the 2nd flush (day 56). After removing the casing, compost was mixed manually for 3 min, after which samples were frozen in liquid nitrogen and stored at −20 °C.

### Transformation of *A. bisporus*

The coding sequence of *mnp1* (gene ID 221245) was synthesized at Genscript (Nanjing, China) with its *Apa*I*, Bsa*I*, Mfe*I*, Bcl*I*, Nco*I*, EcoR*I, and *Bcl*I restriction sites removed (Additional file 1: Text 1) without altering the amino acid sequence (Additional file 1: Text 2). The actin promoter and terminator were amplified from genomic DNA of *A. bisporus* A15 using Taq polymerase and primers 1 & 2 and 3 & 4, respectively (Additional file 1: Table S1). Fragments were cloned in pUC20 using *Hin*dIII*/Nco*I, and *Bam*HI*/Eco*RI, respectively. The *mnp1* coding sequence was cloned in between the *actin* regulatory elements using *Nco*I and *Bam*HI. The *mnp1* coding sequence flanked by the actin regulatory elements was amplified using Phusion Hot Start II High-Fidelity DNA polymerase (Thermo Fisher Scientific, Waltham, USA) and primers 5 & 6 (Additional file 1: Table S1). The PCR product was cloned in pBHgPA digested with *Pac*I and *Sgs*I using InFusion cloning (Pelkmans et al. 2016). The resulting plasmid pBHg-221245-ActPT was introduced in *A. bisporus* A15 gills using *Agrobacterium tumefaciens* strain AGL-1 (Chen et al. 2000b). Transformants were screened on MEA plates containing 25 µg ml^−1^ hygromycin, 200 µM cefotaxime, and 100 µg ml^−1^ chloramphenicol, and transferred to a second selection plate containing 40 µg ml^−1^ hygromycin.

### Laccase and manganese peroxidase activity

Compost samples were taken up in 10 volumes demineralized water and shaken at 250 rpm at 25 °C for 1 h. The extract was separated from insoluble particles at 15,000 g for 15 min. Lac activity was determined by mixing 10–100 µl five times diluted compost extract with 1 mM 2,2′-azino-bis(3-ethylbenzothiazoline-6-sulphonic acid) in 1 ml citric phosphate buffer pH 4. Change in absorbance at 420 nm was followed for 30 s. MnP activity was measured via the oxidative coupling of 3-methyl-2-benzothiazolinone hydrazone (MBTH) and (3-dimethylamino)benzoic acid (DMAB) (Castillo et al. 1994). The reaction mix contained 0.07 mM MBTH, 0.99 mM DMAB, 0.1 mM MnSO_4_, 0.05 mM H_2_0_2_, and 100 mM succinic-lactate buffer pH 5. The reaction was followed for 30 s at 590 nm. Compost extract was diluted to obtain changes in OD <0.3 (usually five times diluted extract was used). The Mn^2+^ independent activity was obtained by adding 1 mM 2,2′,2′′,2′′′-(Ethane-1,2-diyldinitrilo)tetra-acetic acid (EDTA). MnP activity was defined as the difference in OD change over time in the presence and absence of EDTA. Lac and MnP activities were expressed in units (U) using the law of Lambert–Beer with an extinction coefficient of 36,000 and 53,000 M^−1^ cm^−1^, respectively.

### Chitin assay

N-acetylglucosamine (GlcNAc) release from chitin was determined as described (Vos et al. 2017). In short, homogenized lyophilized compost (see below) was treated with KOH, followed by an incubation with chitinase and lyticase. OD_585_ was converted to [GlcNAc] using a reference line of 0–300 µM GlcNAc.

### Lignin analysis with pyrolysis-GC–MS

Lyophilized compost (15 g) was ground using a mortar and homogenized using a Tissuelyser and grinding jar (Qiagen, Hilden, Germany) at 30 Hz for 1 min. Technical triplicates of 60–70 and 100–107 µg compost were used for pyrolysis of wheat straw and PII, and PIII and PIV samples, respectively. Samples were pyrolyzed at 500 °C for 1 min with helium as carrier gas using a 2020 microfurnace pyrolyzer (Frontier Laboratories, New Ulm, MN, USA) with an AS 1020E Autoshot GC–MS, using a Trace GC with a DB1701 fused-silica capillary column coupled to a DSQ-II (El at 70 eV). Amdis software was used to characterize peaks in the GC–MS chromatogram. Molar areas were calculated by dividing areas by the molecular weight of the corresponding molecule (Jurak et al. 2015).

### Total carbohydrate content and composition

Composition and content of neutral carbohydrates in homogenized compost samples (see above) was determined in technical duplicates using gas chromatography with inositol as an internal standard (Englyst et al. 1982). Glucose, arabinose, rhamnose, xylose, galactose, and mannose were used as standards with a quantity of 1 mg each. Samples were hydrolyzed with 1 M H_2_SO_4_ for 3 h at 100 °C after treatment with 72% (w/w) H_2_SO_4_ for 1 h at 30 °C. Sugar monomers were derivatized to their alditol acetates by reducing the sugars with sodium borohydride followed by acetylation of the formed alditols and analysed using a Focus-GC (Thermo Scientific, Waltham, MA, USA). Uronic acid content was determined as the anhydro-uronic acid content using a *m*-hydroxydiphenyl assay in which Na_2_B_4_O_7_ was added. Analysis was performed using an autoanalyser (Skalar Analytical, Breda, The Netherlands) with 12.5–100 μg ml^−1^ glucuronic acid as a standard (Thibault 1979). Total carbohydrate content was calculated as the sum of the neutral carbohydrate and uronic acid content.

### Enzymatic saccharification and monosaccharide quantification

Technical duplicates of 250 mg homogenized compost samples (see above) were taken up in 9.75 ml 50 mM NaOAc buffer (pH 5) containing 0.25 mg ml^−1^ NaN_3_ by vortexing. Samples were heated at 100 °C for 10 min, followed by addition of 0.25 ml of a mix of cellulases and hemicellulases (Cellic Ctec2, 2.7% w/w of DM and Cellic Htec, 0.3% w/w of DM). The enzyme preparations were kindly provided by Novozymes (Bagsvaerd, Denmark). After rotation head over tail for 24 h at 50 °C, samples were heated at 100 °C for 10 min. Insoluble particles were removed by centrifugation at 20 °C for 10 min at 10,000*g*. As a control, samples were incubated without enzymes. Content of monosaccharides was analysed by high performance anion exchange chromatography using a Dionex ICS-5000 unit (Dionex, Sunnyvale, CA, USA) equipped with a CarboPac PA-1 column (2 mm × 250 mm ID) in combination with a Carbopac guard column (2 mm × 50 mm ID) and pulsed amperometric detection. Chromelion software (Thermo Scientific, Sunnyvale, CA, USA) was used to control the system. Elution was performed with a flow rate of 0.4 ml^−1^ min as follows: 40 min 100% H_2_O; 5 min from 100% 1 M NaOH to 100% 1 M NaOAc; 5 min 100% 1 M NaOAc; 8 min with 100% H_2_O; followed by elution for 15 min at 0.1 ml min^−1^ using 0.5 M NaOH. Monosaccharides were detected and quantified during the first 40 min and the last 15 min of elution. Calibration curves of 12.5–100 μg ml^−1^ rhamnose, galactose, glucose, xylose and mannose were used as a reference.

### Hydrogen peroxide consumption and production

The H_2_O_2_ consuming and producing capacity were measured in compost extract using a Hydrogen Peroxide Assay Kit (BioVision, Milpitas, CA, USA). The *de novo* H_2_O_2_ production in compost extract was calculated using measurements 8, 13, 18, 23, and 28 min after addition of horseradish peroxidase (HRP) and OxiRed. In order to determine H_2_O_2_ consumption in compost the compost extract was supplemented with 10 µM H_2_O_2_. The H_2_O_2_ consumption was calculated based on the remaining H_2_O_2_ in compost extract after incubation at room temperature for 10, 20, 30, and 40 min and corrected for H_2_O_2_ production. A reaction volume contained 25 µl compost extract, 25 µl 40 µM H_2_O_2_ and 50 µl reaction mix containing 0.4 µl HRP and 0.2 µl OxiRed. A standard curve of 0–12.8 µM H_2_O_2_ was used to calculate H_2_O_2_ producing and consuming capacity in nmol min^−1^ g^−1^ compost.

### Data analysis

Differences between strains and temporal changes in H_2_O_2_ production and consumption were assessed with a T test using SPSS 22. Temporal changes in other variables were assessed using ANOVA followed by a Bonferroni or Dunnett’s T3 post hoc correction. Ratios of S/G, Ox/non-Ox, and Ph-1,2/Ph-3 were log transformed prior to testing. Statistical significance is indicated with *(p < 0.05), **(p < 0.01), and ***(p < 0.001).

## Results

**Fig. 1:**
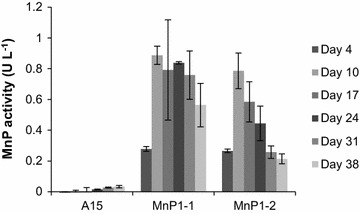
MnP activity of A15, MnP1-1, and MnP1-2 in malt extract. *Error bars* indicate standard deviation of biological triplicates

**Fig. 2:**
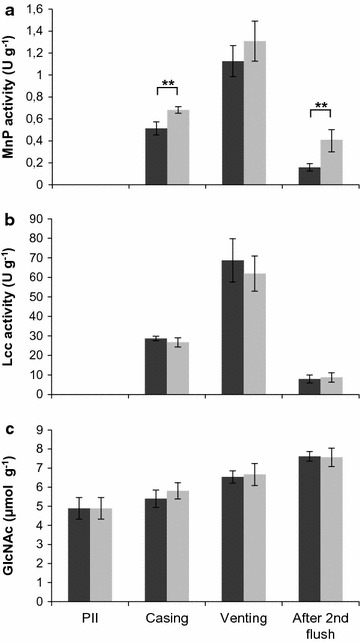
MnP (**a**) and Lcc (**b**) activity and GlcNAc release (**c**) in a semi-commercial cultivation of A15 (*dark grey shading*) and the MnP overexpressor MnP1-1 (*light grey shading*). Significant differences between A15 and MnP1-1 at each time point are indicated. Significant differences between time points are presented in Additional file 1: Table S2. *Error bars* represent standard deviation of biological quadruplicates

**Fig. 3:**
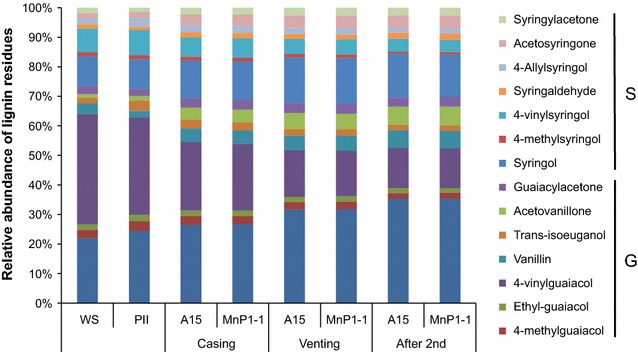
Relative abundance of lignin derived residues as measured by pyrolysis-GC/MS in compost samples of a semi-commercial cultivation of A15 and the MnP overexpressor MnP1-1. Each compost sample represents the average of biological quadruplicates. *Error bars* have been left out for clarity, all values are available in Additional file 1: Table S3. *WS* wheat straw, *PII* compost from end of phase II

**Fig. 4:**
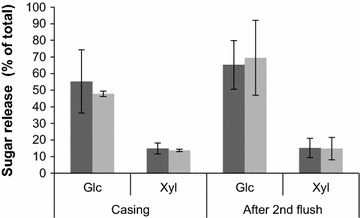
Glucose (Glc) and xylose (Xyl) release after treating compost at casing and after the 2nd flush with cellulases and hemicellulases. Monosaccharide release is expressed as a percentage of the total corresponding carbohydrate. *Error bars* represent standard deviations based on four biological replicas

Gene *mnp1* of *A. bisporus* cloned in between the actin regulatory sequences was introduced in *A. bisporus*. Transformants MnP1-1 and MnP1-2 produced 0.9 and 0.8 U l^−1^ MnP activity after 17 days of growth on malt extract, respectively (Fig. 1), while wild type strain A15 did not produce activity. A semi-commercial cultivation was performed with strain A15 and MnP1-1. Lac activity (Fig. 2b) and chitin content (Fig. 2c) did not differ between the strains throughout cultivation. In addition, flush pattern and mushroom yield was not affected (data not shown). In contrast, MnP activity g^−1^ wet compost was increased 0.30- and 3-fold at casing (day 15) and the 2nd flush (day 56), respectively (Fig. 2a). Pyrolysis-GC/MS showed that the ratio of syringyl to guaiacyl derived residues (S/G) in compost colonized with A15 or MnP1-1 had increased from 0.38 (PII-end) to 0.47–0.48 at venting (Table 1; Fig. 3) and decreased to 0.43 after the 2nd flush. The relative abundance of oxidized to non-oxidized lignin derived residues (Ox/non-Ox) was also not different between the strains with values of 0.28, 0.47, 0.72, and 0.93 at PII, casing, venting, and after the 2nd flush in the case of the wild-type, respectively. The relative abundance of lignin derived residues of the phenylmethane and phenylethane type relative to those of the phenylpropane type (Ph-C1,2/Ph-C3, del Rio et al. 2002) decreased in compost colonized by A15 from 5.46 (PII-end compost) to 4.17 (venting). After the 2nd flush, Ph-C1,2/Ph-C3 had increased to 4.53, a similar value to casing. In compost colonized by MnP1-1, Ph-C1,2/Ph-C3 decreased similarly to A15. However, after the 2nd flush Ph-C1,2/Ph-C3 of compost colonized by MnP1-1 had not increased and differed significantly from the Ph-C1,2/Ph-C3 after the 2nd flush of A15 (T-test after log transformation, p < 0.05 after Bonferroni correction). With a total of 30–35% of the lignin being removed after the 2nd flush, as based on pyrolysis and Klason lignin content, no differences were found between A15 and MnP1-1 (not shown). Moreover, carbohydrate accessibility and composition were similar. Hydrolysis of the carbohydrates in the compost showed that total carbohydrate content (w/w %) decreased by about 50% from PII-end to the 2nd flush in case of A15 and MnP1-1 (Table 2). Moreover, glucose and xylose release after incubating compost with cellulases and hemicellulases was similar between A15 and MnP1-1 (Fig. 4).

**Table 1:** S/G, Ox/non-Ox, and Ph-C1,2/Ph-C3 ratios in compost colonized by strain A15 or MnP1-1

	A15			MnP1-1		
	S/G^a^	Ox/Non-ox^b^	Ph1,2/Ph3^b^	S/G^b^	Ox/Non-ox^b^	Ph1,2/Ph3^b^
PII-end	0.38 (0.028) AB	0.29 (0.01) A	5.46 (0.153) A	0.38 (0.028) A	0.29 (0.01) A	5.46 (0.153) A
Casing	0.45 (0.024) AB	0.47 (0.038) B	4.68 (0.074) B	0.46 (0.019) BC	0.49 (0.033) B	4.59 (0.202) B
Venting	0.48 (0.002) A	0.73 (0.013) C	4.17 (0.04) C	0.48 (0.013) B	0.73 (0.023) C	4.15 (0.088) C
2nd flush	0.44 (0.005) B	0.94 (0.023) D	4.53 (0.046) B*	0.43 (0.017) C	0.97 (0.026) D	4.35 (0.053) BC*

Each value represents the average of four biological replicas except for PII-end which represents three replicas and standard deviations are shown in brackets. Means of each ratio that share a letter (A, B or C) are not significantly different (ANOVA after log transformation). Differences between A15 and MnP1-1 are indicated with an asterisk (*) (T-test, Bonferroni correction, p < 0.01)

^a^ANOVA with Dunnett’s T3 post hoc test

^b^ANOVA with Bonferroni post hoc test

**Table 2:** Carbohydrate composition, degree of substitution, and total carbohydrate content of compost colonized by A15 or strain MnP1-1

Strain	Sample	Carbohydrates (mol %)^a^							Degree of substitution		w/w %^b^
		Rha	Ara	Xyl	Man^c^	Gal	Glc^d^	UA	Ara/Xyl	UA/Xyl	Total
	WS	0.5 (0.3)	5.2 (0.2)	38.2 (1)	0.6 (0.1)	1.4 (0.1)	50.0 (1)	3.9 (0.6)	13.7 (0.3)	10.2 (1.7)	60.2 (1.4)
	PII-end	0.6 (0.2)	4.4 (0.1)	32.7 (1.7)	1.7 (0)	1.5 (0.1)	53.8 (3.4)	5.3 (2.2)	13.5 (0.6)	15.9 (6.1)	30.9 (3.6)
A15	Casing	0.7 (0.2)	4.4 (0.3)	32.3 (2.4)	3.1 (0.3)	1.7 (0.2)	50.9 (4.1)	6.9 (1.3)	13.7 (0.7)	21.1 (2.7)	21.8 (4.1)
	Venting	0.5 (0.1)	4.7 (0.5)	30.1 (3)	10.5 (9.3)	1.8 (0.5)	44.3 (9.5)	8.1 (2)	15.6 (2.8)	27.4 (8.2)	21.3 (4)
	After 2nd flush	0.9 (0.2)	6.3 (0.3)	26.4 (1.6)	10.1 (5.6)	2.5 (1.3)	41.7 (4.9)	12.0 (2)	24.1 (2.4)	45.8 (9.4)	12.0 (1.1)
MnP1-1	Casing	0.6 (0.1)	4.3 (0.2)	31.5 (1.2)	3.1 (0.6)	1.6 (0.1)	52.4 (1.4)	6.5 (1.1)	13.7 (1.1)	20.7 (2.9)	24.1 (1.5)
	Venting	0.7 (0.3)	4.4 (0.6)	28.6 (5.5)	13.6 (12)	1.6 (0.2)	43.7 (10.3)	7.4 (1.5)	15.7 (3.4)	27.2 (10.6)	25.2 (4.6)
	After 2nd flush	1.1 (0.3)	5.8 (0.1)	24.5 (2)	10.9 (6.2)	2.6 (0.7)	44.0 (5.6)	11.1 (1.9)	23.9 (1.9)	45.8 (11.1)	13.0 (0.7)

Standard deviation is shown in parentheses. *Rha* rhamnosyl, *Ara* arabinosyl, *Xyl* xylosyl, *Man* mannosyl, *Gal* galactosyl, *Glc* glucosyl, *UA* uronyl residues

^a^Ratio mol/100 mol

^b^Based on dry matter

^c^Not corrected for mannitol

^d^Not corrected for sorbitol and trehalose

**Fig. 5:**
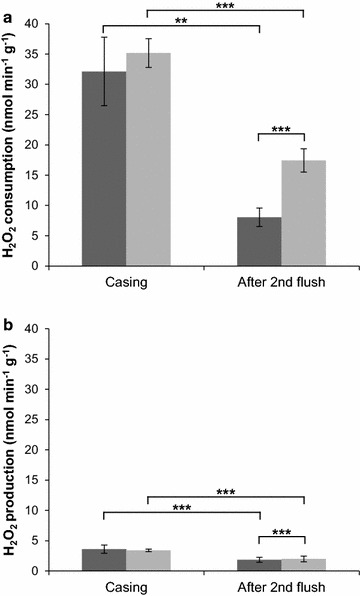
H_2_O_2_ consumption and production in compost extract from a semi-commercial cultivation of A15 (*dark shading*) and MnP1-1 (*light shading*). Statistical difference between the time points and between A15 and MnP1-1 are indicated. *Error bars* represent standard deviations based on biological quadruplicates

Consumption and production of the MnP co-factor H_2_O_2_ in compost extract was monitored during cultivation of A15 and MnP1-1 (Fig. 5). The H_2_O_2_ consuming capacity of A15 compost extract was 32 and 8 nmol min^−1^ g^−1^ wet compost at casing and after the 2nd flush, respectively. The consuming capacity in compost extract of MnP1-1 was similar to that of A15 at casing but was twofold higher with 17.5 nmol min^−1^ g^−1^ wet compost after the 2nd flush. H_2_O_2_ producing capacity in compost extract of A15 was 3.6 and 1.85 nmol min^−1^ g^−1^ wet compost, respectively, at casing and after the 2nd flush. The H_2_O_2_ producing capacity in extract from MnP1-1 was higher after the 2nd flush with 1.99 nmol min^−1^ g^−1^ wet compost. Together, consuming capacity of H_2_O_2_ was always 4- to 8-fold higher than its production, indicating that the generation of co-factor H_2_O_2_ is limiting MnP activity in compost colonized by A15 and MnP1-1.

## Discussion

A significant fraction of carbohydrates is not utilized by *A. bisporus* during colonization and mushroom production (Iiyama et al. 1994; Chen et al. 2000a; Kabel et al. 2017). This may in part be explained by limited degradation of lignin that makes the carbohydrates inaccessible to enzymatic degradation (ten Have et al. 2003). *A. bisporus* removes about 50% of the lignin during PIII, while an additional 8% is removed during PIV (Kabel et al. 2017). This correlates with MnP activity during these phases (Bonnen et al. 1994). Here, the *mnp1* gene was over-expressed using *actin* regulatory sequences with the aim to increase MnP activity during vegetative growth and mushroom production; thus increasing lignin degradation, and, as a consequence, carbohydrate utilization by *A. bisporus.* Transformants MnP1-1 and MnP1-2 produced MnP activity in malt extract, while strain A15 did not. Moreover, MnP was overproduced 3- to 4-fold in wheat bran (data not shown) and a 40% increase in compost was observed in transformants MnP1-1 and MnP1-2 relative to A15 in a small scale cultivation (data not shown). MnP activity of MnP1-1 relative to A15 was increased 0.3- and 3-fold at casing and 2nd flush, respectively, in a large scale cultivation. However, no major changes in lignin content and composition were observed during cultivation of these two strains. The S/G ratio changed from 0.38 in PII-end compost to 0.47 and 0.43 at venting and after the 2nd flush, respectively. This pattern agrees with previous reports (Zeng et al. 2011; Jurak et al. 2015; Patyshakuliyeva et al. 2015). However, ligninolytic activity in other systems has been associated with a decrease rather than an increase of the S/G ratio (Camarero et al. 2001; Martinez et al. 2001; Vane et al. 2001a, 2001b; del Rio et al. 2002; Geib et al. 2008). It is assumed that a higher redox potential and condensation degree makes G lignin more recalcitrant than S lignin. From this perspective, ligninolytic action should result in a decrease of the S/G ratio. In the case of *A. bisporus* the increase in the S/G ratio during casing and venting may relate to a part of the G lignin that is solubilized during PI, PII and/or PIII. This is supported by the observation that the S/G ratio of lignin in water insoluble compost particles at the end of PIII is similar to that of PI, i.e. lower than the S/G ratio in the total PIII sample (Jurak et al. 2015). The Ox/non-Ox ratio increased throughout the cultivation from 0.28 in PII-end compost to 0.93 at the end of the 2nd flush and the Ph-C1,2/Ph-C3 ratio decreased from 5.46 in PII-end compost to 4.17 at venting. The changes in S/G, increase in Ox/non-Ox, and decrease in Ph-C1,2/Ph-C3 after venting show that lignin is modified during PIV. The direction of change of the S/G and Ph-C1,2/Ph-C3 ratio reverted after venting (i.e. a decrease in S/G after venting as compared to an increase during PIII and an increase in Ph-C1,2/Ph-C3 as compared to a decrease during PIII). From this it is concluded that *A. bisporus* affects lignin differently in PIV as compared to PIII, specifically during mushroom formation. This is associated with an increase in cellulase and hemicellulose activity and a decrease in ligninolytic activity during fruiting (Wood and Goodenough 1977; Bonnen et al. 1994; Patyshakuliyeva et al. 2015). Notably, the Ph-C1,2/Ph-C3 ratio had increased after the 2nd flush for compost colonized by A15 but not for compost colonized by MnP1-1. This may be explained by an increased loss of Ph-C1 and Ph-C2 residues as compared to A15 or a higher turnover of Ph-C3 to Ph-C1 and Ph-C2. However, this did not result in detectable differences in carbohydrate composition or accessibility. In contrast, overexpression of a VP in *P. ostreatus* increased the mineralization of lignin and carbohydrate digestibility in cotton stalk (Salame et al. 2012). This illustrates that in other systems ligninolytic action can be improved by overexpression of lignin-modifying enzymes.

The most important co-factors of MnPs are H_2_O_2_ and Mn^2+^. High Mn^2+^ concentration in PIV compost has a positive effect on mushroom yield (Weil et al. 2006). The Mn^2+^ concentration in commercial compost is not limiting MnP activity. Indeed, lignin degradation by A15 or MnP1-1 was not affected by supplementation of Mn^2+^ (data not shown). We did show that the H_2_O_2_ consuming capacity of compost extract of A15 and Mnp1-1 was 4- to 8-fold larger than its producing capacity, implying that this co-factor is limiting for MnP activity and thus explaining why over-expression of *mnp1* does not impact lignin and carbohydrate utilization. H_2_O_2_ as a limiting factor for ligninolytic activity has also been found in *Phanerochaete chrysosporium* (Buswell et al. 1984; Kirk et al. 1986). This makes H_2_O_2_ generation a target for optimizing lignin removal from compost in *A. bisporus* and, consequently, improving carbohydrate consumption of this mushroom forming fungus.

## Additional file

**Additional file 1: Table S1.** Primers used in this study. **Table S2.** Statistical differences over time in MnP and Lcc activity and chitin content in compost as presented in Fig. 2. Time points sharing a letter are not significantly different (ANOVA with Bonferroni post hoc test, p < 0.05). **Table S3.** Relative abundance of lignin residues as percentage of total molar area as presented in Fig. 3. Standard deviations are indicated between brackets.** Text 1**. Nucleotide sequence of the adapted mnp1 over-expressed in* A. bisporus*.** Text 2**. Amino acid sequence of adapted mnp1 over-expressed in* A. bisporus*.

## Abbreviations

DMAB: (3-dimethylamino)benzoic acid
EDTA: 2,2,2,2-(ethane-1,2-diyldinitrilo)tetra-acetic acid
G: guaiacyl
GC: gas chromatography
GlcNAc: N-acetylglucosamine
H: *p*-hydroxyphenyl
HRP: horseradish peroxidase
Lac: laccase
Lip: lignin peroxidase
MBTH: 3-methyl-2-benzothiazolinone hydrazine
MEA: malt extract agar
MnP: manganese peroxidase
MS: mass spectrometry
Non-Ox: non-oxidized
Ox: oxidized
PI: phase I
PII: phase II
PIII: phase III
PIV: phase IV
Ph-C1,2: phenylmethane and phenylethane
Ph-C3: phenylpropane
RH: relative humidity
S: syringyl
VP: versatile peroxidase
U: unit
WB: wheat bran
