# Supplementary material for: H2O2 as a candidate bottleneck for MnP activity during cultivation of Agaricus bisporus in compost
Source: AMB Express. 2017 Jun 17;7:124. doi: 10.1186/s13568-017-0424-z (PMC5474230; doi:10.1186/s13568-017-0424-z)
Supplement: Supplementary file 1 — Additional file 1: Table S1. Primers used in this study. Table S2. Statistical differences over time in MnP and Lcc activity and chitin content in compost as presented in Fig. 2. Time points sharing a letter are not significantly different (ANOVA with Bonferroni post hoc test, p < 0.05). Table S3. Relative abundance of lignin residues as percentage of total molar area as presented in Fig. 3. Standard deviations are indicated between brackets. Text 1. Nucleotide sequence of the adapted mnp1 over-expressed in A. bisporus. Text 2. Amino acid sequence of adapted mnp1 over-expressed in A. bisporus. [file 13568_2017_424_MOESM1_ESM.pdf]

Additional File 1

AMB Express

H_2O_2 as a candidate bottleneck for MnP activity during cultivation of *Agaricus bisporus* in compost

Aurin M. Vos[1], Edita Jurak[2], Jordi F. Pelkmans[1], Koen Herman[1], Gill Pels[2], Johan J. Baars[3], Ed Hendrix[3], Mirjam A. Kabel[2], Luis G. Lugones[1], Han A. B. Wösten[1]*

[1]Microbiology, Department of Biology, Utrecht University, Padualaan 8, 3584 CH Utrecht, The Netherlands; [2]Wageningen University, Laboratory of Food Chemistry, Bornse Weilanden 9, 6708 WG, Wageningen, The Netherlands; [3]Plant Breeding, Wageningen University and Research Centre, 6700 AJ Wageningen, The Netherlands

*Corresponding author
Prof. dr. Han A.B. Wösten
Department of Microbiology, Utrecht University
Padualaan 8, 3584 CH Utrecht
The Netherlands
Telephone: 31 30 2533448
Fax: 31 30 2513655
E-mail: h.a.b.wosten@uu.nl

Table S1: Primers used in this study.

Primer	Primer name	Sequence
**1**	Actin prmtr F	AAGCTTAGCCGAGAGAAGATGCCCC
**2**	Actin prmtr R	CCATGGTTTGTTATTCGTGTGTTCG
**3**	Actin trmntr F	GGATCCGCTGATGGTGCTTTATGATAAATAAAGTCCTTGGG
**4**	Actin trmntr R	GAATTCTACTACTACCCCCAAAACCGACATCATCC
**5**	Act-Pr_F_infu	CCAGGGGGATCGTTAAAGCTTAGCCGAGAGAAG
**6**	Act-Ter_R_infu	CGCCGAATTGGCGCGGAATTCTACTACTACCCCC

Table S2: Statistical differences over time in MnP and Lcc activity and chitin content in compost as presented in Figure 2. Time points sharing a letter are not significantly different (ANOVA with Bonferroni post hoc test, $p < 0.05$).

	A15			MnP1-1		
	**MnP**	**Lcc**	**Chitin**	**MnP**	**Lcc**	**Chitin**
**PII**	-	-	A	-	-	A
**Casing**	A	A	AB	A	A	AB
**Venting**	B	B	BC	B	B	BC
**After 2nd flush**	C	C	C	C	C	C

Table S3: Relative abundance of lignin residues as percentage of total molar area as presented in Figure 3. Standard deviations are indicated between brackets.

		G-residues							
		**Guaiacol**	**4-methylguaiacol**	**Ethyl-guaiacol**	**4-vinylguaiacol**	**Vanillin**	**Trans-isoeuganol**	**Acetovanillone**	**Guaiacylacetone**
	**WS**	22 (4)	2.8 (0.5)	1.9 (0.5)	37.4 (6.4)	3.5 (0.7)	2 (0.5)	1.2 (0.3)	2.8 (0.6)
	**PII**	24.5 (1.4)	3.3 (0.3)	2.1 (0.4)	32.9 (2.9)	2.3 (0.2)	3.4 (0.2)	1.7 (0.2)	2.3 (0.2)
**Casing**	**A15**	26.6 (5.4)	2.9 (0.8)	1.9 (0.6)	23.2 (6.2)	4.6 (1)	2.9 (1)	4.2 (0.7)	3.1 (0.8)
	**MnP1-1**	26.7 (5.6)	2.8 (0.7)	1.9 (0.4)	22.4 (5)	4.6 (0.9)	2.8 (0.6)	4.3 (0.9)	3.1 (0.6)
**Venting**	**A15**	31.7 (3.6)	2.5 (0.4)	1.7 (0.3)	15.9 (1.9)	4.9 (0.6)	2.2 (0.3)	5.4 (0.5)	3.3 (0.4)
	**MnP1-1**	31.9 (1.6)	2.5 (0.2)	1.8 (0.2)	15.5 (1)	5 (0.4)	2.2 (0.2)	5.3 (0.3)	3.3 (0.2)
**After 2nd**	**A15**	35.3 (2.9)	2 (0.2)	1.7 (0.2)	13.6 (1.1)	6 (0.4)	1.7 (0.1)	6.2 (0.3)	3.1 (0.2)
	**MnP1-1**	35.3 (2.8)	2 (0.2)	1.6 (0.2)	13.4 (1.5)	6 (0.8)	1.8 (0.3)	6.4 (0.8)	3.3 (0.4)

		S-residues						
		**Syringol**	**4-methylsyringol**	**4-vinylsyringol**	**Syringaldehyde**	**4-Allylsyringol**	**Acetosyringone**	**Syringylacetone**
	**WS**	10 (1.8)	1.3 (0.3)	8.1 (1.5)	1.4 (0.2)	2.1 (0.4)	1.8 (0.4)	1.8 (0.3)
	**PII**	10.2 (1.5)	1.3 (0.1)	8.5 (1.5)	1.2 (0.2)	3 (0.3)	2 (0.1)	1.5 (0.2)
**Casing**	**A15**	12.8 (2.6)	1.2 (0.3)	6.6 (1.3)	1.6 (0.3)	2.6 (0.5)	3.7 (0.7)	2.1 (0.4)
	**MnP1-1**	13.2 (2.8)	1.3 (0.3)	6.5 (1.3)	1.7 (0.3)	2.6 (0.5)	3.8 (0.7)	2.3 (0.4)
**Venting**	**A15**	15.4 (1.9)	1.1 (0.2)	5.2 (0.6)	1.7 (0.2)	2 (0.2)	4.3 (0.4)	2.6 (0.3)
	**MnP1-1**	15.6 (1.1)	1.2 (0.1)	5.1 (0.5)	1.6 (0.1)	2 (0.1)	4.4 (0.3)	2.6 (0.2)
**After 2nd**	**A15**	14.8 (1.4)	0.8 (0.1)	4.4 (0.2)	2 (0.2)	1.7 (0.1)	4.2 (0.3)	2.6 (0.2)
	**MnP1-1**	14.3 (1.7)	0.8 (0.1)	4.2 (0.6)	2.1 (0.2)	1.7 (0.2)	4.5 (0.5)	2.6 (0.3)

Text 1: Nucleotide sequence of the adapted *mnp1* over-expressed in *A. bisporus*.

CCATGGCTTTCAAAATTCTTCTCAGCCTTATTTTGGCTTTGAACGCCGTCCAATTTATTGCTGGTAC
GCACTTTACTCATATCCTCATATTATTTGCCGACAAGTCAACGATGTAGCTGTTCCTACCAGGAGG
GCGCAATGTGCCGATGGCACCACTGTTTCTAATGAGGCCTGTTGCGTTCTGCTCCCCATTATAGCA
GATATCCAGCCGAACTTGTTCGAAAACGAGTGCGGTGAAGAGGTAATGATTTAATTTTTTGATAGA
GTTACGATATTCAACAGCATATTTATACTTTAGGTCCATGAAACCTTGCGGGCATCATTTCATGATG
CTATCGGATTCTCCAGAGCAGCTGGGTAGGTTCTGACTGTCTGTGGCGGCAGCTAGTATCGCTAAA
ATGATTTCCAGTGGTGGTGGTGCTGATGGTTCTCTCGTCACTTTCGGCGATGTGGAGACGACATTT
GCCGCTAACGCAGGAATCGACGAGATTGTAGAGACATTACGACCTTTTATAAACAGCCACAACAT
CAGTGCCGGCGATTTGTGAGCCTGGACTCATAATCTGTGTTGCATCCTGTTAACGCTCTTCCTAGCA
TCCAGTTTGCAACCGTTGTTGGATTGACCAACTGCCCCGGCGCACCTCGCATACCCTTTTTCTTAGG
CCGCCCAGACGCTACCGCCGCTTCCCCCGATGGTCTTGTCCCTGAGCCCTTCGGTCAGTCTCTCATC
AGCCTTTCCGGTATAATCTACAACCCAAGTTGATAATCAACAGATTCCGTCACGAAAATTCTTGAA
CGCTTCGATGATGCTGGTTTTACCCCCACTGAAGTTGTTGCATTGCTTGCATCGTGAGTCTCTAGAA
CAAGATCGAGAAATATGTGTCCAATCCCCCCGTAGTCATACTGTCGCAGCTTCGGACACTATTGAG
CCCGGTGTAAGTTTATTTCTTCGTCAGTATGCATTCTCATCTCATGAGCACCTTATTCCAGCTCGAA
GGGTAAGACATTCCCTATCAATAAGAAACGTCTCAGTATCCAACGCAACCTCTAGTGTCCCCTTCG
ACTCGACTCCCGGAGAATTTGACAGGCAGTTCTTTATCGAGACTATGCTTAAAGGCACTTCCTTCC
CAGGGTAAGTAGTTCGTTGACATTCGTTACGCATCCTAATTTTGGCAAAGCACTGGGGGGAACCAA
GGTGAAGCCTTATCTCCCTTGCCGGGAGAACTTCGTCTCGAATCCGATGGACTTGTACGTATACAG
TCTTTTGTTGTCTGAATTTGTGATTGTTAACATTATTTATCATTAGTTGGCTCGAGGTCTGTCTTGCT
AACAAAAGGCATCTGATAACATTGAAGGACTAACTTCATCATAGATGAACGAACCGCATGTGACT
GGCAACTCTTCGCTAGTAAGAATGTTTGCCAACCAAGAAGAATAGCTCACCTAAAATTCCACGATA
GCAGATCAACAAAAAATGGCATCGGCATTTAGCGACGCGATGGTCAAACTGTCTCTCGTCGGGCA
AGATAAGAGCCAGTTGATTGATTGTTCAGACGTCATTCCGCGAACGATCCCTTTGACAAATGAACC
ATACTTCCCTGCCGACTTGACCAAAGATGATCTTGAGCAGACCGTGAGTCCAATAGACATTCACAA
TACGATTTCCACTCTGAGCCTGCTTGTTTTTGATACAGTGCCCGGATGAATTTCCTGATTACCCTTC
CAACCCCAGTGTCACTTCGGTCGCGCCAGTGTAAGTTTATGAACAGCTCCAGACGTCTGCTCTTAT
TCACTCTGCGCAAATATCTAGTCCCACCTCGTAAGGATCC

Text 2: Amino acid sequence of adapted *mnp1* over-expressed in *A. bisporus*.

M A F K I L L S L I L A L N A V Q F I A A V P T R R A Q C A D G T T V S N E A C C V L L P I I A D I Q
P N L F E N E C G E E V H E T L R A S F H D A I G F S R A A G G G G A D G S L V T F G D V E T T F A
A N A G I D E I V E T L R P F I N S H N I S A G D F I Q F A T V V G L T N C P G A P R I P F F L G R P D
A T A A S P D G L V P E P F D S V T K I L E R F D D A G F T P T E V V A L L A S H T V A A S D T I E P
G L E G V P F D S T P G E F D R Q F F I E T M L K G T S F P G T G G N Q G E A L S P L P G E L R L E S
D G L L A R D E R T A C D W Q L F A T D Q Q K M A S A F S D A M V K L S L V G Q D K S Q L I D C
S D V I P R T I P L T N E P Y F P A D L T K D D L E Q T C P D E F P D Y P S N P S V T S V A P V P T S
